# Simultaneous bilateral femoral neck fractures in a dialysis-dependent patient: case report and literature review

**DOI:** 10.1186/s12891-020-03281-7

**Published:** 2020-04-15

**Authors:** Yunyun Zhu, Jingtao Hu, Wenlun Han, Jianwei Lu, Yuqing Zeng

**Affiliations:** 1 Department of Nephrology, Tongde Hospital of Zhejiang Province, HangZhou, Zhejiang Province China; 2 Zhejiang Chinese Medical University, HangZhou, Zhejiang Province China; 3 Department of Orthopaedics, Tongde Hospital of Zhejiang Province, 234 GuCui Road, HangZhou, 310012 Zhejiang Province China

**Keywords:** Femoral neck fractures, Bilateral, Dialysis, Osteoporosis, Case report

## Abstract

****Background**:**

Simultaneous bilateral femoral neck fractures are extremely rare without obvious injury. Herein, we report the case of a patient on dialysis presenting with bilateral femoral neck fractures, which is a condition with high complication and mortality rates according to a review of the pertinent literature.

****Case presentation**:**

We report the case a 47-year-old female with a history of 8 years of haemodialysis due to polycystic kidney disease who presented with bilateral hip pain during walking. The clinical history and results of physical and radiographic examinations of this patient are shown. Single-stage bilateral hemiarthroplasty was performed after a multidisciplinary team consultation. Three days after the operation, she could ambulate with a walker. The woman gradually regained her previous ability to walk over 6 months after surgery.

**Conclusions:**

A multidisciplinary team consultation for perioperative management is necessary and effective in patients on dialysis. Early diagnosis with prompt surgical treatment could lead to favourable recovery.

## Background

Renal osteodystrophy (RO) secondary to chronic kidney disease (CKD) is an established cause of pathological femur neck fractures [1]. The incidence of hip fracture in patients undergoing haemodialysis (HD) is significantly higher than that in the general population. The incidence of hip fracture in dialysis patients is 4.4 times higher than that of the general population [2], and its incidence is 29.3 / 1000 people / year [3]. However, simultaneous bilateral femoral neck fractures (SBFNFs) in patients on dialysis are extremely uncommon [4]. We report a rare case of SBFNFs in a middle-aged patient on dialysis and review SBFNFs in patients with CKD.

## Case presentation

**Fig. 1:**
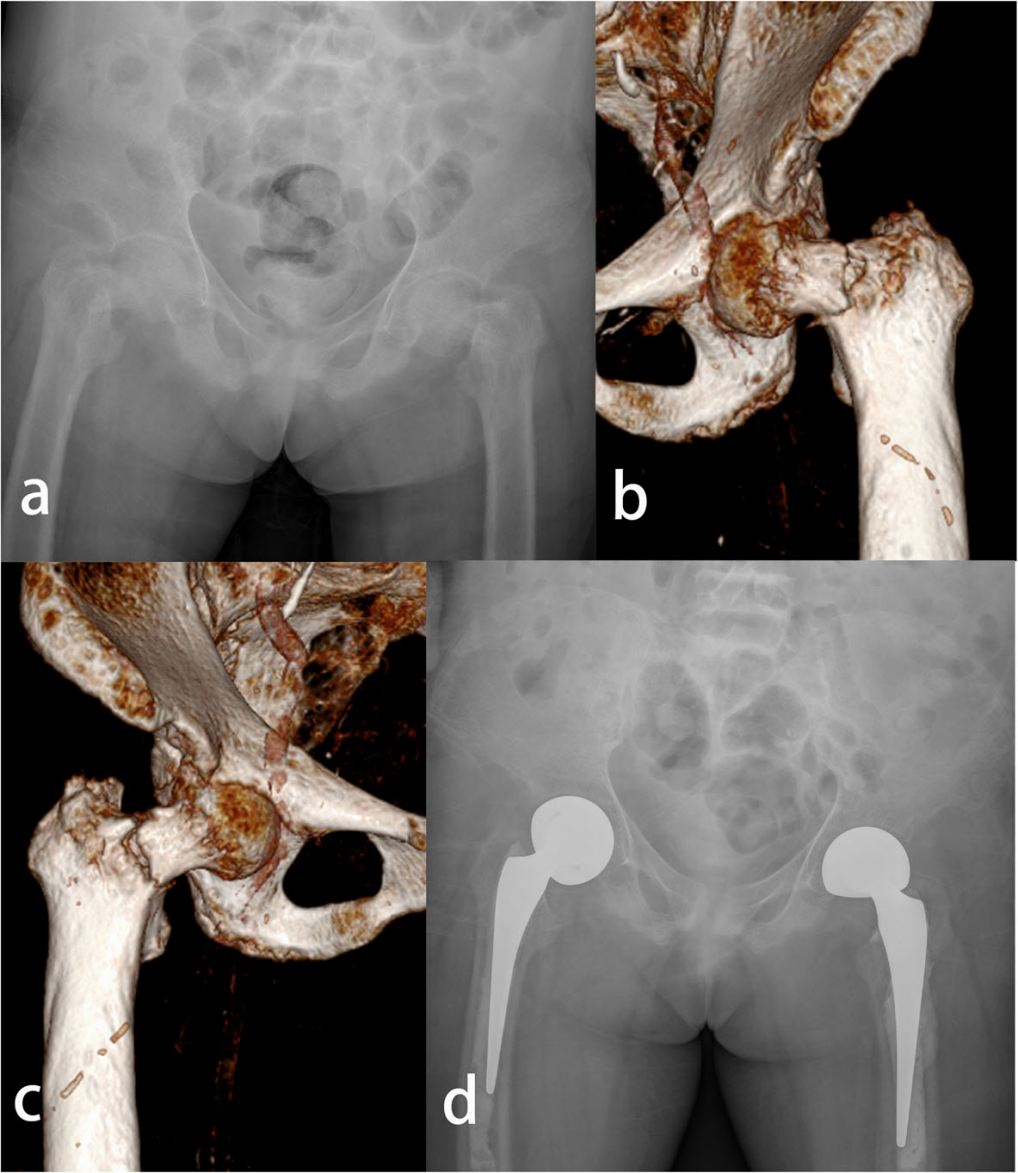
**a**: Hip X-ray showing bilateral femoral displaced fractures with low bone density. **b**: CT scan showing the femoral displaced fracture on the left side. **c**: CT scan showing the femoral displaced fracture on the right side. **d**: Bilateral femoral displaced fractures treated with bilateral hip hemiarthroplasty

The patient was a 47-year-old female with no history of trauma who developed bilateral hip pain 1 day prior to admission. She had a history of haemodialysis due to polycystic kidney disease. Upon physical examination, she demonstrated inability to move the hip joints and bilateral inguinal tenderness. Laboratory studies showed anaemia (haemogram 8.5 g/dL), a low normal albumin value (3.1 g/dL), a high parathyroid hormone level (907 pg/ml), hypocalcaemia (5.1 mg/dL), hyperphosphatemia (2.3 mmol/L), and elevated alkaline phosphatase activity (1228 U/dL). Hip X-ray (Fig. 1a) and CT (Fig. 1b, Fig. 1c) examinations showed bilateral femoral displaced fractures. Bone mineral density testing revealed osteoporosis (T = 3.0). She also had a history of hyperparathyroidism secondary to CKD and parathyroidectomy.

We promptly performed a multidisciplinary team consultation to make preoperative preparations and performed the surgery 4 days after admission. Considering her mobility and life expectancy, we performed concurrent bilateral hip hemiarthroplasty with cement prostheses (Fig. 1d). The operative findings included severe osteoporosis and displaced femoral neck fractures. Anti-osteoporosis medication was administered after surgery. Meanwhile, the patient continued maintenance haemodialysis. Three days after the operation, she could ambulate with a walker. The follow-up appointments at 3 and 6 months revealed that the woman had gradually regained her previous ability to walk.

## Discussion and conclusions

SBFNFs in patients on dialysis are relatively rare. To the best of our knowledge, only 10 cases have been reported previously, making our case the 11th (Table 1) [1, 5–14]. SBFNFs are usually secondary to seizure disorders [15], trauma [16], electric shock injuries [17], hypovitaminosis D [18], osteoporosis [19], and metabolic diseases. Stress fractures of the bilateral femoral neck occasionally occur in young patients [20]. It has been reported that the incidence of hip fractures in the general population has decreased significantly [21]. In the contrary, the incidence of hip fractures among haemodialysis patients is increasing [22, 23]. However, in Japan, compared to the general population, in Japanese dialysis patients, the incidence of hip fractures decreased among women but did not change among men between 2008 and 2013 [24]. Although the incidence of hip fractures appears to have decreased slightly, it remains a challenging problem in dialysis patients for both orthopaedic surgeons and nephrologists [25].

**Table 1:** Summary of all studies in the English literature reporting bilateral pathological neck femur fractures in chronic renal disease

No	Reference	Year	No. of cases	SEX	Age	Primary renal	Treatment	Dialysis (Y/N)
1	﻿Zingraf et al. [5]	1974	1		45	Unknown	Arthroplasty	Y
2	Gerster et al. [6]	1983	1 out of 2 cases reported had CRF	Case 1: F Case 2: F	Case 1: 69 Case 2: 78	Case 1: CP Case 2: Unknown	Case 1: bilateral THR Case 2: Conservative for right hip and Osteosynthesis for left intertrochanteric hip	Case 1: N Case 2: N
3	Ogun et al. [7]	2001	2	Case 1: M Case 2: M	Case 1:45 Case 2:35	Case 1: amyloidosis Case 2: nephrolithiasis	Osteosynthesis with multiple cannulated screws in both cases	Case 1: N Case 2: N
4	Hung et al. [8]	2001	1	F	39	Unknown	Bilateral hemiarthroplasty	N
5	Karapinar et al. [1]	2003	1	M	23	Obstructive uropathy	Bilateral cemented THA	Y
6	Devkota et al. [9]	2013	1	F	47	DN and HN	Non-operative management	Y
7	Garcia et al. [10]	2014	1	F	43	Reflux nephropathy	Osteosynthesis	Y
8	Satyanarayana et al. [11]	2015	1	M	23	Reflux nephropathy	Bilateral, uncemented, modular bipolar hemiarthroplasty	Y
9	Mazzola et al. [12]	2015	1	F	76	Unknown	right cemented hemiarthroplasty and left fixation with dynamic hip screw	Y
10	Freitas et al. [13]	2016	1	F	49	Unknown	Bilateral total hip arthroplasty	Y
11	John et al. [14]	2018	3	case1:M case2: F case3.M	case1:44 case2:15 case3.64	PKD CDK CP	Osteosynthesis Osteosynthesis Conservative (infected)	case1:Y case2:Y case3.Y
12	current study	2020	1	F	47	PKD	bilateral hip hemiarthoplasty with cement prosthesis concurrently	Y

*Abbreviations*: *F* female, *M* male, chronic pyelonephritis *CP* congenital dysplastic kidneys *CDK*, *DN* diabetic nephropathy, *HN* hypertensive nephropathy, *PKD* polycystic kidney disease

Among end-stage renal disease (ESRD) patients, HD is associated with a 61% higher risk of hip fracture than peritoneal dialysis (PD) [26]. Nevertheless, PD, HD, and kidney transplantation (KT) patients and HD and KT patients had the highest and lowest risk of hip fractures, respectively [27]. One study found that an advanced age, low body weight, low serum albumin level, and high alkaline phosphatase (ALP) and parathyroid hormone levels were associated with a low bone mass in HD patients [28]. The patient in our case had a parathyroid hormone level of more than 5000 pg/ml until undergoing parathyroidectomy. Thus, we suggest thatmaintaining an adequate body weight and serum albumin level, regular monitoring of the femoral neck bone mineral density, and undertaking an exercise programme are important to improve bone health in patients on HD.

Guidelines recommend that surgery for hip fracture should be performed within 48 h after the event. Delayed surgery for more than 48 h in elderly patients with hip fractures increased the risk of postoperative complications. However, in dialysis patients, a delayed operation did not contribute significantly to the mortality rate compared to that in the non-dialysis cohort [29]. The current study found that delaying surgery for a period of time did not negatively impact the incidence of postoperative complications [30].

﻿Hip fractures are a common problem in the ageing population and are associated with significant mortality and morbidity rates. The mortality rate within 1 year after hip fracture is as high as 36% despite aggressive management, including surgery and rehabilitation [31]. Unfortunately, the incidence, mortality, and medical costs of fractures in patients with kidney disease are much higher than those of ordinary fractures [32, 33]. Patients with CKD often have a longer hospital stay after a fracture, and the chance of going to a skilled nursing facility after discharge is higher than that of patients without CKD. According to reports, more than 80% of fracture patients with CKD need skilled nursing facility after discharge form the hospital, which is much higher than other complications of CKD [34].

Femoral neck fractures are often treated with either internal fixation or artificial hip replacement. In the general population, treatment is performed according to age, bone quality, and fracture classification. In our case, we performed hemiarthroplasty according to the poor bone quality and life expectancy of less than 10 years. In the patient dependent on dialysis, the outcomes of arthroplasty are well described in the recent literature. Compared to non-dialysis-dependent patients, dialysis patients undergoing arthroplasty have suboptimal results with significantly higher incidence rates of deep venous thrombosis, surgical site infection, need for blood transfusions, wound complications, intensive care unit care, and attentive postoperative rehabilitation, as well as a 10–20 times greater risk of inpatient mortality [35, 36]. Some authors have suggested that arthroplasty should be approached with caution and preferably be delayed until after KT [37, 38]. In our case, blood transfusions were performed, and a seizure occurred 2 weeks after surgery. There are two possible factors may contribute to the high mortality in dialysis patients with hip fracture. The first is the higher prevalence of comorbidities and the second is the higher occurrences of postoperative complications. A personized surgical treatment based on specific clinical situations should be planned and carried out using a necessary risk assessment before surgery [39]. It should be noted that biological and mechanical failure can occur in the patients with RO and osteoporosis after hip fracture surgery [40]. Thus, a team approach involving expert nephrologists, geriatricians and orthopaedic surgeons is highly significant to reduce early complications and mortality. Dialysis patients with hip fracture require multidisciplinary assessment and intervention for the prevention of subsequent osteoporotic fractures. It can be difficult for dialysis patients to participate in rehabilitation while undergoing dialysis. Therefore, clinicians should highly pay attention to their standard rehabilitation.

In conclusion, hip fractures in HD patients pose serious challenges for surgeons. Clinicians should pay more attention to comprehensive treatment methods, which can reduce the morbidity and mortality rates of hip fractures in HD patients. A multidisciplinary team consultation for perioperative management is necessary and effective. Early diagnosis with prompt surgical treatment could lead to favourable recovery.

## Data Availability

The datasets used and analysed during the current study are available from the corresponding author on reasonable request.

## Abbreviations

RO: Renal osteodystrophy
HD: Haemodialysis
CKD: Chronic kidney diseases
SBFNF: Simultaneous bilateral neck femoral fractures
PD: Peritoneal dialysis
KT: Kidney transplantation

## References

[1] Karapinar H, Ozdemir M, Akyol S, Ulku O (2003). Spontaneous bilateral femoral neck fractures in a young adult with chronic renal failure. Acta Orthop Belg.

[2] Alem AM, Sherrard DJ, Gillen DL, Weiss NS, Beresford SA, Heckbert SR (2000). Increased risk of hip fracture among patients with end-stage renal disease. Kidney Int.

[3] Nair SS, Mitani AA, Goldstein BA, Chertow GM, Lowenberg DW, Winkelmayer WC (2013). Temporal Trends in the Incidence , Treatment , and Outcomes of Hip Fracture in Older Patients Initiating Dialysis in the United States. Clin J Am Soc Nephrol.

[4] Yassin A, Jawad I, Coomber R, Gonzalez-Castro A. Non-traumatic, bilateral subcapital femoral fractures postpartum. BMJ Case Rep. 2014;2014. 10.1136/bcr-2013-201625 PMC3902394 24445846

[5] Zingraff J, Drueke T, Roux JP, Rondon-Nucete M, Man NK, Jungers P (1974). Bilateral fracture of the femoral neck complicating uremic bone disease prior to chronic hemodialysis. Clin Nephrol.

[6] Gerster JC, Charhon SA, Jaeger P, Boivin G, Briancon D, Rostan A (1983). Bilateral fractures of femoral neck in patients with moderate renal failure receiving fluoride for spinal osteoporosis. Br Med J (Clin Res Ed).

[7] Ogun TC, Memik R, Yel M, Sarlak A (2001). Bilateral pathologic femoral neck fracture as a consequence of renal osteodystrophy: report of two cases and review of the literature. Artroplasti Artroskopik Cerrahi.

[8] Hung KH, Lee CT, Gau YL, Chen JB (2001). Neglected bilateral femoral neck fractures in a patient with end-stage renal disease before chronic dialysis. Ren Fail.

[9] Devkota P, Ahmad S (2013). Bilateral impacted femoral neck fracture in a renal disease patient. Niger Med J.

[10] Garcia FL, Dalio RB, Sugo AT, Picado CH (2014). Bilateral spontaneous fracturing of the femoral neck in a patient with renal osteodystrophy. Rev Bras Ortop.

[11] Sathyanarayana V, Patel MT, Raghavan S, Naresh D (2015). Simultaneous bilateral femur neck fracture in a young adult with chronic renal failure- a case report and review of literature. J Orthop Case Rep..

[12] Mazzola P, Anzuini A, Picone D, De Filippi F, Dubner L, Bellelli G (2015). Simultaneous bilateral femoral neck fracture and end-stage renal disease in a 76-year-old woman: a case report. Aging Clin Exp Res.

[13] Freitas A, de Macedo Neto SL, Loures FB, Neto EDS, de Alencar Barreto LC, Camilo MS (2016). Simultaneous bilateral femoral neck fracture in a patient with renal osteodystrophy. Trauma Case Rep.

[14] John R, Kumar P, Aggarwal S, Rajnish RK, Agarwal S, Vatsyan K (2018). Simultaneous, non-traumatic, bilateral neck femur fractures in uremic renal Osteodystrophy: a report of three cases and literature review. J Orthop Case Rep.

[15] Brennan SA, O'Neill CJ, Tarazi M, Moran R (2013). Bilateral neck of femur fractures secondary to seizure. Pract Neurol.

[16] Gao YS, Zhu ZH, Zhang CQ (2015). Simultaneous bilateral fractures of the femoral neck caused by high energy: a case report and literature review. Chin J Traumatol.

[17] Sohal HS, Goyal D (2013). Simultaneous bilateral femoral neck fractures after electrical shock injury: a case report. Chin J Traumatol.

[18] Paraliticci G, David Rodriguez-Quintana R, Davila A, Otero-Lopez A (2015). Atraumatic bilateral femoral neck fractures in a premenopausal female with hypovitaminosis D. Bol Asoc Med P R.

[19] Emami MJ, Abdollahpour HR, Kazemi AR, Vosoughi AR (2012). Bilateral subcapital femoral neck fractures secondary to transient osteoporosis during pregnancy: a case report. J Orthop Surg (Hong Kong).

[20] Jasqui-Remba S, Jasqui-Bucay Alan, Jasqui-Bucay Ariel, Fernandez-De-Lara-Barrera Y. Bilateral femoral neck stress fractures in a high-performance young female runner. BMJ Case Rep. 2019;12(8). 10.1136/bcr-2019-230900 PMC6721772 31451476

[21] Wright NC, Saag KG, Curtis JR, Smith WK, Kilgore ML, Morrisey MA (2012). Recent trends in hip fracture rates by race/ethnicity among older US adults. J Bone Miner Res.

[22] Wagner J, Jhaveri KD, Rosen L, Sunday S, Mathew AT, Fishbane S (2014). Increased bone fractures among elderly United States hemodialysis patients. Nephrol Dial Transplant.

[23] Arneson TJ, Li S, Liu J, Kilpatrick RD, Newsome BB, St Peter WL (2013). Trends in hip fracture rates in US hemodialysis patients, 1993-2010. Am J Kidney Dis.

[24] Wakasugi M, Kazama JJ, Wada A, Hamano T, Masakane I, Narita I (2018). Hip fracture trends in Japanese Dialysis patients, 2008-2013. Am J Kidney Dis.

[25] Denburg M, Nickolas TL (2018). Declining hip fracture rates in Dialysis patients: is this winning the war?. Am J Kidney Dis.

[26] Boonpheng B, Thongprayoon C, Mao MA, Wijarnpreecha K, Bathini T, Kaewput W (2019). Risk of hip fracture in patients on hemodialysis versus peritoneal dialysis: a meta-analysis of observational studies. J Evid Based Med.

[27] Tan J, Li Y, Wu Z, Zhao J (2018). Risk of hip fracture in patients on dialysis or kidney transplant: a meta-analysis of 14 cohort studies. Ther Clin Risk Manag.

[28] Huang GS, Chu TS, Lou MF, Hwang SL, Yang RS (2009). Factors associated with low bone mass in the hemodialysis patients--a cross-sectional correlation study. BMC Musculoskelet Disord.

[29] Swift O, Ayub A, Mathavakkannan S, de Roeck N (2016). Outcomes following surgery for fractured neck of femur in dialysis patients: a 5-year review from a district general hospital in the United Kingdom. BMC Nephrol.

[30] Dong C, Wang Y, Wang Z, Wang Y, Wu S, Du Q (2016). Damage control orthopedics management as vital procedure in elderly patients with femoral neck fractures complicated with chronic renal failure: a retrospective cohort study. PLoS One.

[31] Bhandari M, Devereaux PJ, Swiontkowski MF, Tornetta P, Obremskey W, Koval KJ (2003). Internal fixation compared with arthroplasty for displaced fractures of the femoral neck. A meta-analysis. J Bone Joint Surg Am.

[32] Kim SM, Long J, Montez-Rath M, Leonard M, Chertow GM (2016). Hip fracture in patients with non-Dialysis-requiring chronic kidney disease. J Bone Miner Res.

[33] Beaubrun AC, Kilpatrick RD, Freburger JK, Bradbury BD, Wang L, Brookhart MA (2013). Temporal trends in fracture rates and postdischarge outcomes among hemodialysis patients. J Am Soc Nephrol.

[34] Doan QV, Gleeson M, Kim J, Borker R, Griffiths R, Dubois RW (2007). Economic burden of cardiovascular events and fractures among patients with end-stage renal disease. Curr Med Res Opin.

[35] Patterson JT, Tillinghast K, Ward D (2018). Dialysis dependence predicts complications, intensive care unit care, length of stay, and skilled nursing needs in elective primary Total knee and hip Arthroplasty. J Arthroplast.

[36] Yoo JY, Restrepo C, Maltenfort MG, Parvizi J, Erkocak OF (2016). Incidence of infection and Inhospital mortality in patients with chronic renal failure after Total joint Arthroplasty. J Arthroplast.

[37] Cavanaugh PK, Chen AF, Rasouli MR, Post ZD, Orozco FR, Ong AC (2016). Complications and mortality in chronic renal failure patients undergoing Total joint Arthroplasty: a comparison between Dialysis and renal transplant patients. J Arthroplast.

[38] Ponnusamy KE, Jain A, Thakkar SC, Sterling RS, Skolasky RL, Khanuja HS (2015). Inpatient mortality and morbidity for Dialysis-dependent patients undergoing primary Total hip or knee Arthroplasty. J Bone Joint Surg Am.

[39] Pedersen AB, Christiansen CF, Gammelager H, Kahlert J, Sorensen HT (2016). Risk of acute renal failure and mortality after surgery for a fracture of the hip: a population-based cohort study. Bone Joint J.

[40] Song KS, Yoon SP, Lee SK, Lee SH, Yang BS, Park BM (2017). The results of proximal femoral nail for intertrochanteric fracture in hemodialysis patient. Hip Pelvis.

